# Supramalleolar osteotomy outcomes for post-traumatic fracture-related ankle arthritis: a retrospective analysis

**DOI:** 10.1186/s12893-025-03087-1

**Published:** 2025-08-11

**Authors:** Yun-Qing Zhao, Xue-Wen Wang, Heng Li, Xiao-Feng Gong, Yong Wu

**Affiliations:** 1 Department of Foot and Ankle Surgery, Beijing Jishuitan Hospital, Capital Medical University, No.31, Xinjiekou East Street, Xicheng District, Beijing, 100035 China; 2 Department of Orthopedics, Beijing Puren Hospital, Beijing, 100010 China

**Keywords:** Ankle joint, Joint preservation, Fracture, Clinical efficacy, Realignment surgery

## Abstract

**Background:**

This study aimed to investigate the early-to-mid-term efficacy of supramalleolar osteotomy (SMO) for the management of post-traumatic ankle arthritis resulting from previous fractures.

**Methods:**

A retrospective analysis was conducted on the clinical data of 19 individuals with post-traumatic ankle arthritis secondary to old fractures treated with SMO between March 2018 and September 2022. The cohort included 6 males and 13 females, aged 15 to 59 years, with an average of 37.1 ± 15.8 years. Among these cases, 12 underwent surgical treatment for their initial fractures, while 7 received conservative management. Clinical efficacy was assessed using the American Orthopedic Foot and Ankle Society (AOFAS) ankle-hindfoot score, Foot Function Index (FFI), and Visual Analog Score (VAS) for pain. Imaging analyses were conducted using the changes of tibial anterior surface angle (TAS), talar tilt (TT) angle, and modified Takakura stage. Evaluation of patient satisfaction after surgery was conducted during the final follow-up.

**Results:**

All patients were followed up for 12 to 47 months (mean follow-up: 21.9 ± 11.1 months). At the final follow-up, the AOFAS ankle-hindfoot score improved to 81.9 ± 9.8, the FFI decreased to 24.2 ± 22.8, and the VAS score decreased to 3.2 ± 3.0, all showing significant improvement compared with preoperative values (*p* < 0.05). The TAS angle increased significantly to 90.2° ± 5.1° postoperatively (*p* < 0.05), while the TT angle was 0.3° ± 2.5°, without a statistically significant change (*p* = 0.314). No significant progression was observed in the modified Takakura stage (*p* = 0.458). The overall patient satisfaction rate was 89.5% (17/19). Prognostic analysis revealed no correlation between fracture-to-surgery interval (1–27 years) and AOFAS improvement ( *r* = 0.233, *p* = 0.337). Excessive preoperative TT (> 7.3°) did not compromise outcomes after correction.

**Conclusions:**

SMO demonstrates favorable early-to-mid-term efficacy in the treatment of traumatic fracture-related ankle arthritis, with significant improvements in pain relief, functional scores, and patient satisfaction. Prognostic factors (time since fracture, severe talar tilt) did not adversely affect results, supporting SMO’s utility across diverse presentations.

## Background

Traumatic events account for more than 90% of cases of ankle arthritis [1]. Malunion following perimalleolar fractures, ankle sprains, or epiphyseal injuries (in adolescents) disrupts ankle alignment, accelerates joint degeneration, and causes chronic pain [1–3]. Closed or surgical reduction is often conducted to restore anatomical structures in cases of malleolar fractures, distal tibia-fibula fractures, pilon fractures, and talar fractures, which may involve the ankle joint and subsequently lead to post-traumatic ankle arthritis [2]. Malunion after a fracture can result in deformities in the coronal or sagittal plane of the ankle, widening of the ankle joint space, lateral displacement or tilt of the talus, mismatching of the tibiotalar joint, and changes in the alignment of the lower extremities, often resulting in chronic pain and limited range of motion [1, 4, 5]. In adolescents, epiphyseal injuries after fracture are also a notable cause of traumatic ankle arthritis and, if left untreated, may lead to progressive joint degeneration, necessitating timely intervention [4, 6]. 

For early-to-mid-stage arthritis, joint-preserving procedures such as corrective osteotomy [7], supramalleolar osteotomy (SMO) [8–11], and intra-articular osteotomy [12, 13] are viable options. For advanced-stage ankle arthritis, ankle arthrodesis or arthroplasty is typically more appropriate [14]. SMO, as a crucial component of ankle-sparing surgery, realigns the mechanical axis, redistributes joint forces, and may delay arthritis progression [15, 16]. 

However, critical evidence gaps persist. While SMO outcomes are documented, there are few articles mentioning the efficacy of ankle arthritis secondary to perimalleolar fractures, and the impact of fracture-to-surgery interval and preoperative talar tilt severity on efficacy remains poorly characterized. This study undertook a retrospective analysis of clinical data from patients with ankle arthritis secondary to old fractures who underwent SMO, aiming to evaluate the clinical efficacy of this surgical approach for these patients. Before drawing conclusions, we hypothesize that SMO has a better curative effect for this group of patients, and there is no significant relationship between the surgical curative effect and the time interval from the initial fracture to the current surgery.

## Methods

### (1) Timeframe of the study

This retrospective study analyzed data from a cohort of 35 patients treated with SMO between March 2018 and September 2022 at the Department of Foot and Ankle Surgery, Jishuitan Hospital.

### (2) Database used

Clinical data were extracted from the institutional electronic medical records system, including surgical logs, imaging archives, and follow-up databases.

### (3) Inclusion and exclusion criteria

Inclusion criteria: Patients were included in the study if they met the following conditions: (1) a documented history of distal tibiofibular fracture, ankle fracture, pilon fracture, or epiphyseal injuries (in adolescents) for more than a year. (2) met the diagnostic criteria for ankle arthritis. (3) underwent SMO, including open, closed, or dome osteotomy techniques. (4) provided informed consent for surgery after understanding the surgical plan. (5) possessed complete imaging and follow-up data available for at least one year post-surgery.

Exclusion criteria: patients were excluded if they had any of the following: (1) active ankle infection. (2) Charcot joint disease (3) neuroarthropathy on the affected side. (4) postoperative follow-up duration of less than a year. (5) ankle arthritis classified as modified Takakura stage 4 [17]. 

### (4) Indication for surgery

Surgery was indicated for symptomatic post-traumatic ankle arthritis (persistent pain/limited mobility) with coronal/sagittal plane deformity, Modified Takakura stage 1–3b, and Failure of conservative management.

### (5) Surgical techniques

In this study, the included cases employed various SMO methods, and the selection of specific surgical procedures was often influenced by multiple factors. Firstly, for patients with a tibial articular surface angle (TAS) > 80° and a center of rotation of angulation (CORA) located above the ankle joint, we predominantly used the more convenient medial opening-wedge osteotomy. For patients with severe deformity and a CORA at or below the ankle joint level, when soft tissue conditions permit, we often selected dome osteotomy to achieve a greater degree of deformity correction.

All patients included in this study had a clear history of fracture. During injury or surgical treatment of fractures, the medial and lateral soft tissues of some patients were damaged, with issues such as scarring or poor elasticity. In such cases, we tended to choose the corresponding medial/lateral closing-wedge osteotomy based on the patient’s varus or valgus condition, with the aim of protecting soft tissues as much as possible and reducing complications such as postoperative wound non-healing and skin necrosis.

The patients were administered spinal anesthesia and were then arranged in the supine position. A tourniquet was applied at the root of the thigh and inflated to a pressure of 300 mmHg. The surgical approach was determined based on the osteotomy technique. For a medial opening-wedge tibial osteotomy, the anteromedial approach to the distal tibia was conducted. For a dome osteotomy or lateral closed-wedge distal tibial osteotomy, the anterolateral approach to the distal tibia was used. The skin, subcutaneous tissue, and fascia were incised to expose the anterior malleolus and neck of the talus. The great saphenous vein within the surgical field was carefully dissociated.

Osteophytes on the anterior malleolus, as well as medial and lateral osteophytes, were excised. The center of rotation and angulation point was determined preoperatively using weight-bearing X-ray of ankle joint [12]. Based on this planning, 2 to 4 K-wires were inserted to define the osteotomy plane. The distal tibial osteotomy was performed once the osteotomy positioning line was confirmed to be satisfactory via fluoroscopy. The distal tibia was then distracted, and the tibial anterior surface angle (TAS) was manually corrected. Alignment was verified using C-arm fluoroscopy, and temporary fixation was achieved with a K-wire or distractor.

If a fibular osteotomy was planned before surgery, a longitudinal incision of approximately 2 cm was made on the distal lateral side of the fibula either on the same plane or extended from the original incision. The skin, subcutaneous tissue, and periosteum were incised to expose and protect the superficial peroneal nerve. The plane for the wedge osteotomy was determined using fluoroscopy, based on the preoperative plan. Holes were drilled along the osteotomy line using a K-wire, and the fibular osteotomy was performed obliquely using an osteotome. If fibular fixation was required, the incision was extended proximally and distally as needed to accommodate the plate length (typically 6–8 cm total incision length) and allow direct visualization for screw placement.

If a dome or closed-wedge osteotomy was conducted without bone grafting (12 cases), a steel plate was positioned for fluoroscopic confirmation of proper alignment in all planes. After plate positioning and correction of varus and valgus deformities were verified satisfactorily under fluoroscopy, the surgical incision was sutured to close in layers, completing the procedure.

In cases involving medial or anterior open wedge osteotomies, the osteotomy gap was filled with tibial autologous bone (3 cases), allogeneic bone (3 cases provided by Beijing Xinkangchen Medical Technology Development Co., Ltd., Beijing, China), or a three-dimensional printed metal wedge (1 case) [18]. After releasing the K-wire or distractor, fluoroscopy was repeated to confirm the correction of the TAS with the restoration of the alignment. A steel plate was then applied for fixation(3 cases provided by Beijing Keyibang’en Medical Device Technology Co., Ltd., Beijing, China).

In this group, five patients underwent fibular osteotomy and subsequent fixation, with one case fixed using K-wire and four cases fixed with a plate. The remaining patients did not require fibular fixation.

### (6) Postoperative management

Following surgery, the affected limb was immobilized using a short posterior support plaster cast for a duration of three weeks. At the three-week postoperative follow-up, the plaster cast and the incision sutures were removed in an outpatient clinic. Ankle joint mobilization and muscle function exercises were initiated between four to six weeks postoperatively. Partial weight-bearing was allowed under the protection of an inflatable walking boot after the seven-week outpatient follow-up. X-rays were taken at 12 weeks postoperatively, and if satisfactory fracture healing was observed, patients were allowed to gradually progress to full weight-bearing ambulation.

### (7) Parameters for assessment

Postoperative follow-up visits were scheduled at intervals of three weeks, six weeks, three months, six months, and twelve months. Subsequent annual follow-ups were conducted either in person or via telephone, depending on individual patient circumstances. Functional outcomes and pain levels were assessed using the American Association of Foot and Ankle Surgery (AOFAS) Ankle-Hindfoot Score, Foot Function Index (FFI), and the Visual Analog Score (VAS) for pain at both preoperative and final follow-ups.

**Fig. 1:**
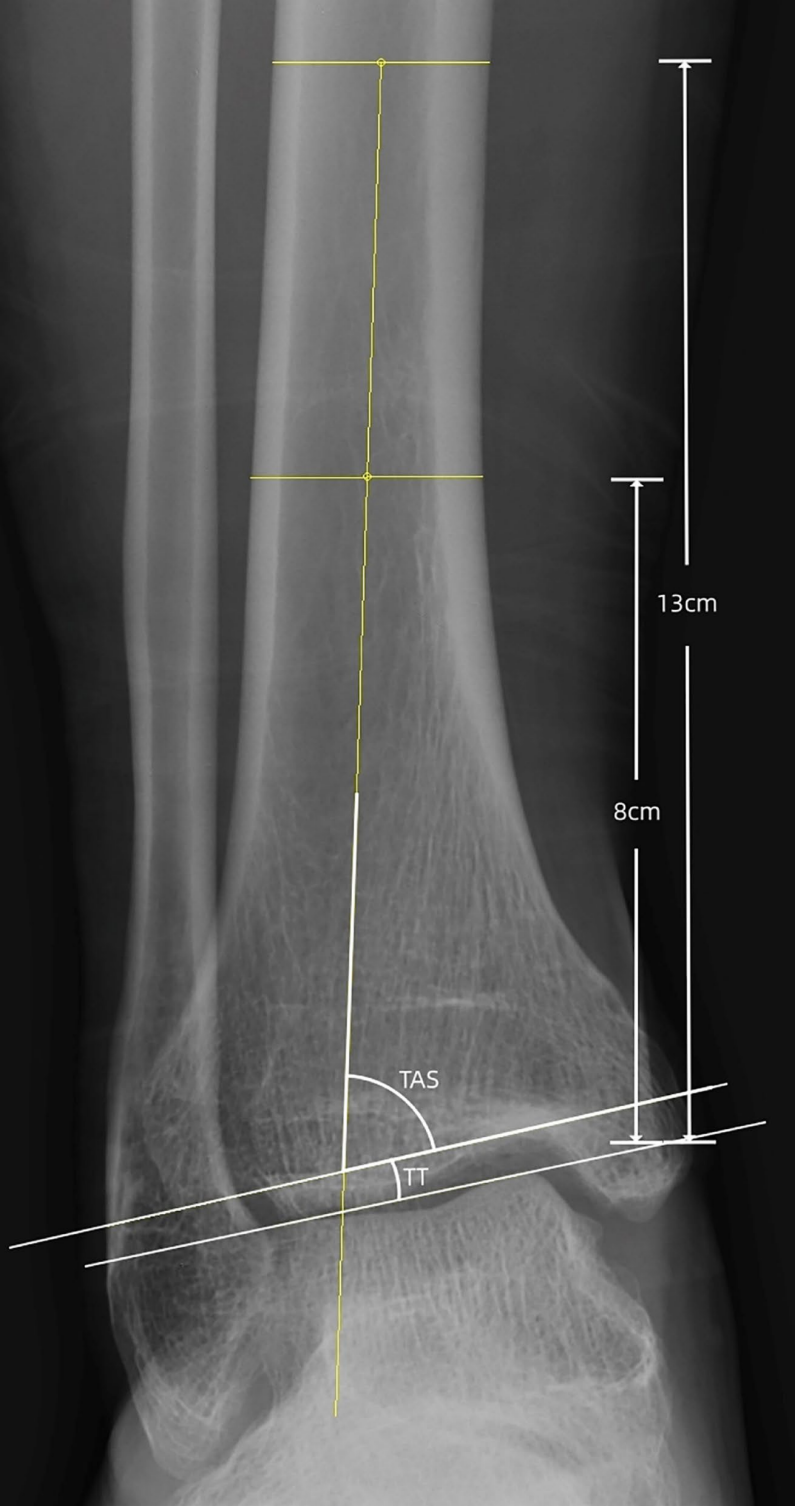
Radiographic parameters. The tibial axis was characterized by a line connecting the midpoints of the tibial shaft at 8 cm and 13 cm proximal to the medial malleolus tip. The angle formed between the distal tibial articular surface and this tibial axis defines the Tibiotalar Surface Angle (TAS). The angle between the superior talar articular surface line and the distal tibial articular surface constitutes the Talar Tilt (TT) angle

The progression of ankle arthritis was evaluated using the modified Takakura staging system. Weight-bearing X-rays of the ankle joint were obtained preoperatively and at the final follow-up to assess alignment correction, specifically measuring the TAS and talar tilt (TT) angle. The tibial axis was characterized by a line connecting the midpoints of the tibial shaft at 8 cm and 13 cm proximal to the medial malleolus tip. In the context of the weight-bearing AP ankle view, the TAS angle is defined as the angle formed between the tibial axis and the distal tibial articular surface. Conversely, the TT angle is represented by the angle between the distal tibial articular surface and a line tangential to the superior surface of the talus. The measurement methodology is depicted in Fig. 1. Postoperative complications and patient satisfaction were systematically recorded and analyzed.

### (8) Statistical analysis

Statistical analyses were performed using SPSS software (version 26.0). Consistent with the method described by Lee et al., modified Takakura staging values were assigned numerical equivalents: stages 3a, 3b, and 4 were designated as 3, 3.5, and 4, respectively, to facilitate statistical analysis [19]. The Shapiro–Wilk normality test was used for the measurement data. Normally distributed variables were expressed as the mean ± standard deviation, while non-normally distributed variables were reported as the median (interquartile range).

Preoperative and postoperative AOFAS Ankle-Hindfoot scores, VAS, and TAS followed a normal distribution and were analyzed using paired t-tests. The FFI, TT, and Takakura staging data, which did not conform to normal distribution, were evaluated using paired Wilcoxon tests. To evaluate the potential impact of the time interval between fracture occurrence and SMO on clinical outcomes, Pearson correlation analysis was performed between (1) the fracture-to-SMO interval (years) and (2) the change in AOFAS ankle-hindfoot score (postoperative minus preoperative value).A significance level of α = 0.05 was applied to all statistical tests.

### (9) Ethical considerations

The hospital’s Ethics Committee approved this study (No: K2022-105-00) and waived the requirement for informed consent due to the retrospective nature of the study.

## Results

After the inclusion and exclusion process, a total of 19 cases were finally included in the study. The patients were followed for at least 12 months postoperatively, with a follow-up period ranging from 12 to 47 months and an average of 21.9 ± 11.1 months. The cohort comprised 6 males and 13 females, with an age range of 15 to 59 years and an average age of 37.1 ± 15.8 years. Among the cases, 5 involved the left ankle, and 14 involved the right. Predominant clinical manifestations included recurrent ankle pain and restricted range of motion. The time interval between the initial fracture and current surgical intervention ranged from 1 to 27 years, with an average of 9.5 ± 8.0 years.

The types of old fractures included distal tibiofibular fractures in 4 cases, ankle fractures in 10 cases, pilon fractures in 4 cases, and a combined ankle and talus fracture in 1 case. A total of 12 cases had undergone surgical treatment following their initial fracture, while the remaining 7 patients had received conservative treatment. Based on the Takakura staging system for ankle arthritis, 2 cases were classified as stage 1, 10 cases as stage 2, 1 as stage 3a, and 3 as stage 3b. In addition, 3 cases were not staged due to blocking by internal fixation (*n* = 1) and valgus ankle arthritis (*n* = 2).

Additional procedures were performed as necessary based on specific clinical conditions. Two patients with lateral collateral ligament injuries underwent ligament repair. Four patients with significant osteophyte hyperplasia underwent either arthroscopic or incisional debridement of the ankle joint. Two patients with gastrocnemius contracture underwent release of the gastrocnemius aponeurosis. Four patients with Achilles tendon contracture underwent Achilles tendon lengthening. One patient with tibia shortening underwent simultaneous tibial lengthening with the assistance of an external fixator. Another patient with an old medial malleolus fracture also underwent reduction and internal fixation for the malleolus fracture. The basic characteristics of the patients are shown in Table 1.

**Table 1:** Clinical data of enrolled patients

No.	Age (years)	Gender	Type of fracture	Time interval between fracture and SMO surgery (years)	Treatment methods for fracture	Follow-up duration (months)	AOFAS Ankle-Hindfoot Scale		SMO surgery	Combined surgery	Takakura Stage		Complications	Satisfaction
							Before surgery	Final follow-up			Before surgery	Final follow-up		
1	57	Female	Distal tibiofibular fracture	19	Conservative treatment	12	61	85	Medial open	Release of gastrocnemius aponeurosis	3a	2	None	Very satisfied
2	24	Male	Ankle joint fracture	20	Surgical treatment	14	83	98	Lateral closure	Tibial lengthening using an external fixator	2	2	None	Very satisfied
3	15	Female	Pilon fracture	1	Surgical treatment	12	65	83	Medial open and intra-articular osteotomy	Repair of lateral collateral ligament	–	–	None	Very satisfied
4	17	Male	Ankle joint fracture	3	Conservative treatment	29	89	95	Lateral closure	None	1	1	None	Very satisfied
5	57	Female	Ankle joint fracture	27	Conservative treatment	12	35	63	Lateral closure	Arthroscopic debridement of ankle joint	2	1	None	Fairly satisfied
6	20	Female	Ankle joint and talus fracture	10	Surgical treatment	16	80	93	Arc-shaped osteotomy	None	2	2	None	Very satisfied
7	35	Female	Pilon fracture	1	Surgical treatment	47	75	70	Lateral closure	None	2	4	None	Very satisfied
8	59	Female	Ankle joint fracture	1	Conservative treatment	27	75	87	Medial open	None	2	1	None	Very satisfied
9	57	Female	Ankle joint fracture	1	Surgical treatment	44	74	87	Medial open and intra-articular osteotomy	Arthroscopic debridement of ankle joint	3b	4	None	Very satisfied
10	35	Male	Ankle joint fracture	16	Surgical treatment	17	65	82	Medial open and anterior open	None	2	2	None	Very satisfied
11	31	Female	Ankle joint fracture	1	Surgical treatment	39	36	69	Medial open	Achilles tendon lengthening	2	2	Poor incision healing and reduced sensation of the forefoot plantar with claw-shaped toe deformity	Moderately satisfied
12	17	Male	Distal tibiofibular fracture	2	Surgical treatment	19	59	84	Lateral closure	Tibialis posterior tendon lengthening and Achilles tendon lengthening	1	1	None	Very satisfied
13	34	Male	Distal tibiofibular fracture	7	Surgical treatment	20	60	78	Arc-shaped osteotomy	Achilles tendon lengthening and Ankle joint release	2	2	None	Very satisfied
14	44	Female	Ankle joint fracture	13	Surgical treatment	17	67	65	Medial open	Arthroscopic debridement of ankle joint	3b	3b	None	Moderately satisfied
15	55	Female	Ankle joint fracture	13	Conservative treatment	12	81	77	Medial open	Open reduction and internal fixation for old medial malleolus fracture	Valgus	Valgus	None	Fairly satisfied
16	16	Male	Distal tibiofibular fracture	5	Conservative treatment	16	85	85	Medial open	Release of gastrocnemius aponeurosis	2	2	Intraoperative screw breakage	Very satisfied
17	42	Female	Pilon fracture	6	Surgical treatment	28	67	79	Medial open	Achilles tendon lengthening	Valgus	Valgus	None	Very satisfied
18	57	Female	Ankle joint fracture	20	Conservative treatment	23	65	87	Medial open	Arthroscopic debridement of ankle joint and Repair of lateral ligament	3b	2	None	Very satisfied
19	32	Female	Pilon fracture	15	Surgical treatment	12	73	90	Arc-shaped osteotomy	None	2	2	None	Very satisfied

**Fig. 2:**
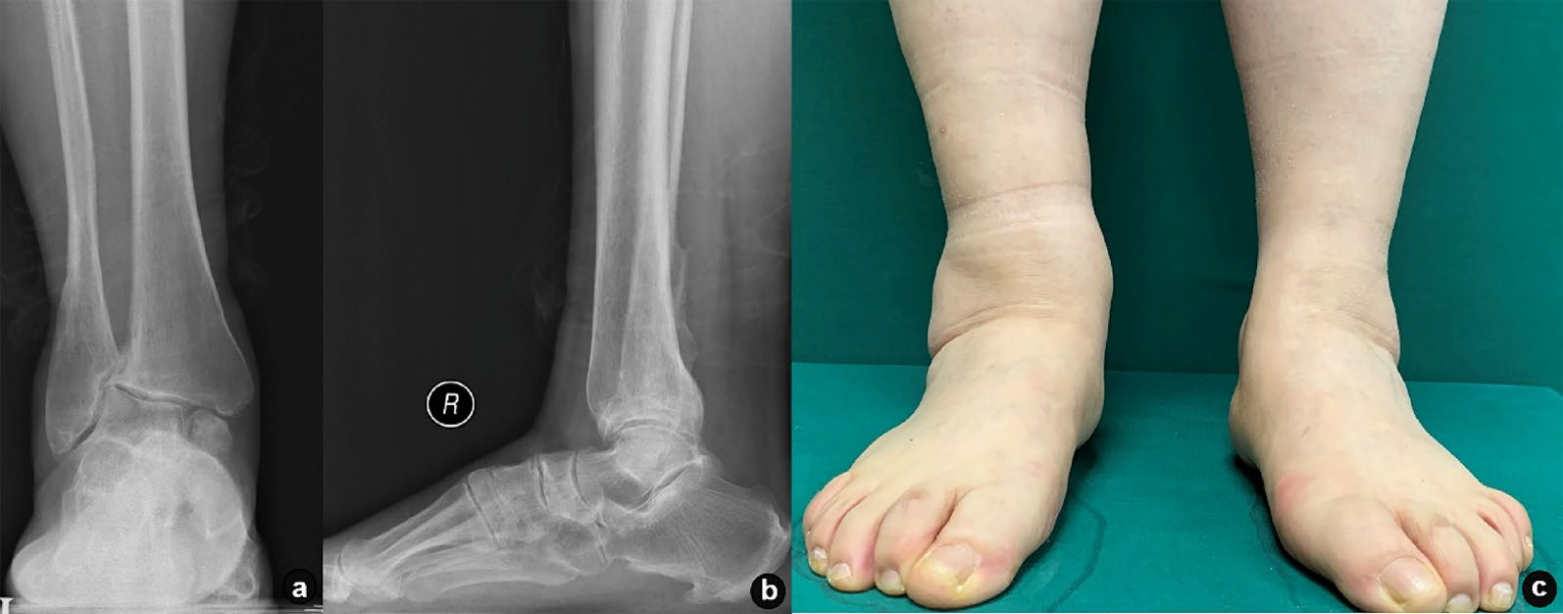
Preoperative radiographs of a specific case. Female, 55 years old, 13 years of conservative treatment for ankle fracture with valgus ankle arthritis. **a**, **b** Preoperative weight-bearing anteroposterior and lateral X-rays of ankle joint; **c** preoperative positioning images showing ankle valgus

**Fig. 3:**
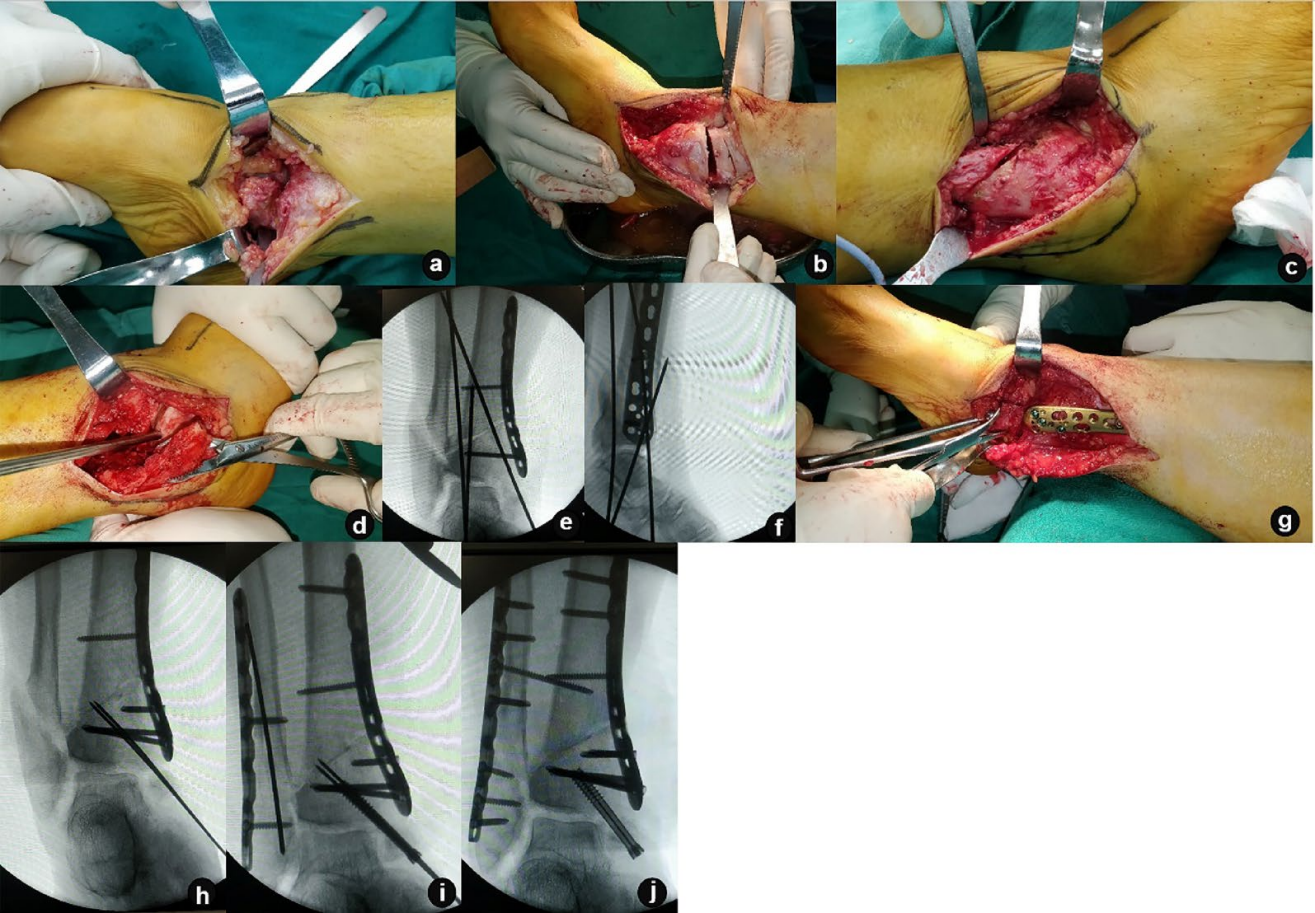
Intraoperative images of the case mentioned. **a**, **b** After the medial incision was made, the scar tissue around the ankle joint was exposed and cleaned, and a medial closed-wedge distal tibial osteotomy was conducted; **c**, **d** A lateral incision was made to verify that fibular fracture site was healed, a fibular osteotomy was conducted proximally to the original fracture line; **e**, **f** After the tibia was fixed with a medial tibial plate, the position was confirmed with satisfaction through anteroposterior and lateral X-rays fluoroscopy; **g**, **h** Reduction and fixation of old fractures of the medial malleolus was conducted with guide pin fixation; **i**, **j** the position was confirmed by fluoroscopy again after medial malleolar screws and plates for lateral malleolar fixation were placed

At the final follow-up, the AOFAS Ankle-Hindfoot Score was 81.9 ± 9.8, the FFI was 24.2 ± 22.8, and the VAS score was 3.2 ± 3.0. These values represented significant improvements from preoperative scores of 68.2 ± 14.5, 50.1 ± 46.5, and 6.1 ± 4.6, respectively. The difference was statistically significant (*p* < 0.05, Table 2). A representative case is illustrated in Figs. 2 and 3.

**Table 2:** Clinical and radiographic outcomes (*n* = 19)

Parameter	PreOP	Last FU	Statistical value	*P* value
AOFAS (points)	68.2 ± 14.5	82.0 ± 9.8	t = −5.561	< 0.001
FFI (points)	50.1 ± 46.5	24.2 ± 22.8	Z = 2.798	0.005
VAS (points)	6.1 ± 4.6	3.2 ± 3.0	t = 3.113	0.006
TAS (SD; degrees)	80.6 ± 9.2	90.2 ± 5.1	t = −5.405	< 0.001
TT (SD; degrees)	1.4 ± 5.4	0.3 ± 2.5	Z = 1.006	0.314
Takakura Classification (1/2/3a/3b/4)	2/10/1/3/0	4/9/0/1/2	Z = 0.742	0.458

Note: AOFAS, the American Orthopedic Foot and Ankle Society ankle-hindfoot scale; FFI, foot function index; VAS, visual analog scale score; TAS, tibial ankle surface angle; TT, talar tilt angle; PreOP, Pre-operative; FU, Follow-Up

**Fig. 4:**
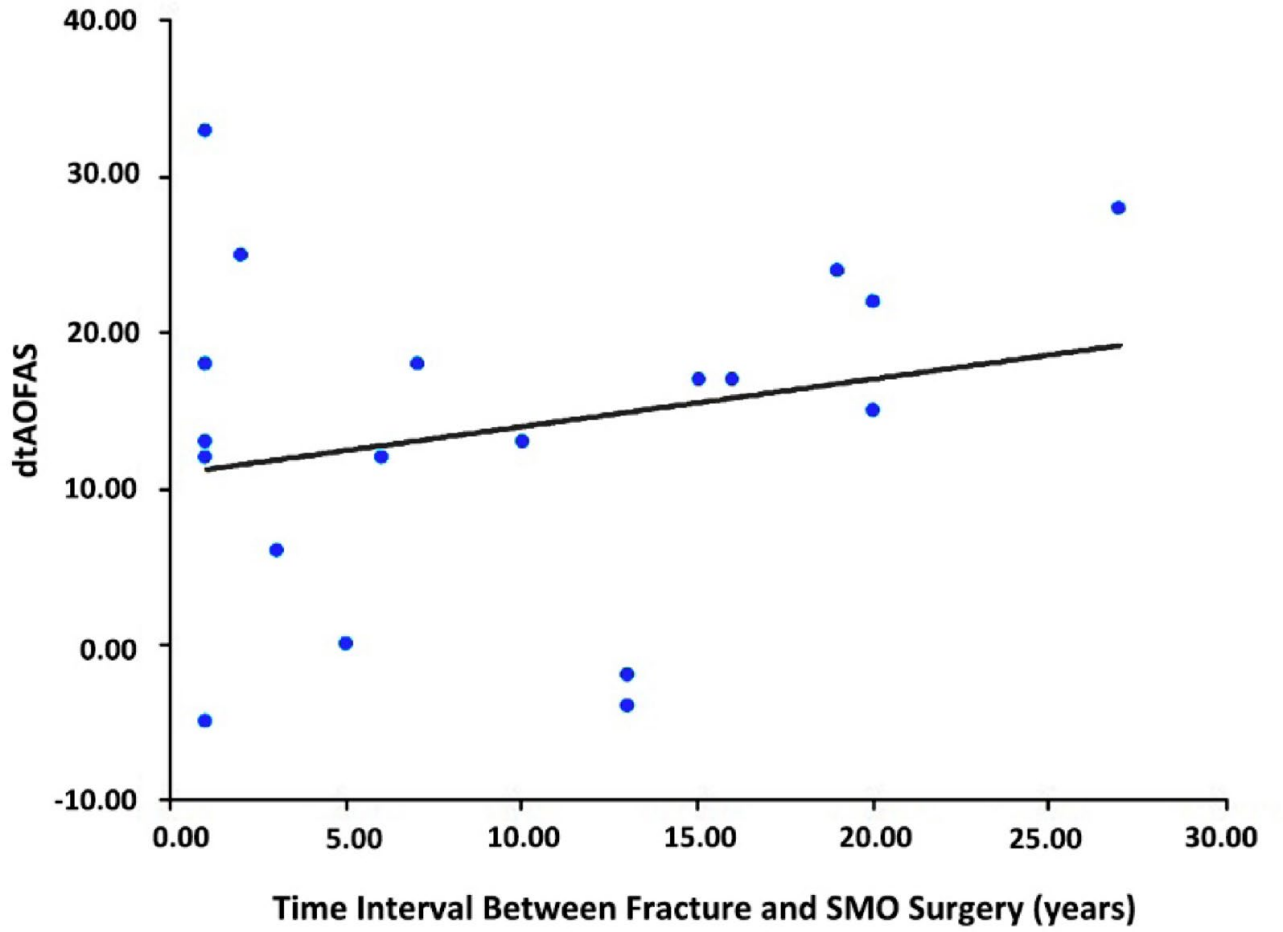
Correlation analysis between clinical efficacy and time interval between fracture and SMO. The time interval between fracture and operation was 1 to 27 years, with an average duration of 9.5 ± 8.0 years. The correlation coefficient between the dependent variable (AOFAS change) and the independent variable (the time interval between fracture and SMO) was 0.233, *p* = 0.337 > 0.05, which was analyzed using Pearson correlation coefficient analysis. There was no correlation between the change of AOFAS before and after surgery and the time interval between fracture and SMO

The imaging results indicated that the mean TAS increased significantly from 80.6° ± 9.2° preoperatively to 90.2° ± 5.1° postoperatively *(p* < 0.05, Table 2). The mean TT angle was 0.3° ± 2.5°, showing no significant decrease compared to the preoperative angle of 1.4° ± 5.4° (*o* = 0.314, Table 2). There was no statistically significant difference in the change of the Takakura staging (*p* = 0.458, Table 2). Furthermore, pearson correlation analysis revealed no significant correlation between the fracture-to-SMO interval (range: 1–27 years; mean: 9.5 ± 8.0 years) and the improvement in AOFAS score (*r* = 0.233, *p* = 0.337) (Fig. 4).

Follow-up radiographs confirmed healing of all osteotomies by three months postoperatively. Two patients developed ankle arthritis during the follow-up period; however, no substantial worsening of clinical symptoms was observed.

Complications: One patient who underwent a medial open wedge osteotomy faced intraoperative screw breakage during the fixation of a 3D-printed metal wedge. This necessitated direct plate fixation without the use of a wedge, autograft, or allograft. The patient achieved satisfactory recovery, and radiographic examination at 4.5 months postoperatively confirmed osteotomy healing and expressed high satisfaction with the surgical outcome.

In another case, a patient who underwent a medial open wedge osteotomy combined with Achilles tendon lengthening developed a postoperative incision infection that failed to heal. After readmission and plate removal seven months postoperatively, the incision healed successfully. The patient complained of reduced medial malleolus pain compared to preoperative levels but experienced increased lateral malleolus pain, ankle joint stiffness, diminished plantar sensation in the forefoot, and claw toe deformity.

Surgical satisfaction: Among the study cohort, 15 patients rated their surgical experience as very satisfactory, 2 as fairly satisfactory, and 2 as moderately satisfactory, resulting in an overall satisfaction rate of 89.5% (17/19).

Among the patients who rated their satisfaction as moderate, one was the patient with the non-healing incision infection. The other patient who underwent medial open wedge osteotomy reported limited pain relief and reduced joint mobility compared to preoperative status. Among patients who expressed satisfaction, all noted significant postoperative pain relief. However, mild restrictions in ankle motion persisted in eight cases.

## Discussion

This study demonstrated favorable early-to-mid-term outcomes of supramalleolar osteotomy (SMO) for post-traumatic ankle arthritis secondary to fracture malunion. Significant improvements were observed in clinical scores (AOFAS, FFI, VAS) and radiographic parameters (TAS angle) at a mean follow-up of 21.9 months. Patient satisfaction was high (89.5%), and complications were minimal. Notably, the progression of arthritis (modified Takakura stage) was halted in most cases. Additionally, our prognostic analysis indicated that neither the interval between fracture and SMO (ranging from 1 to 27 years) nor the severity of preoperative talar tilt (even exceeding 7.3°) adversely affected outcomes. These results confirm SMO’s efficacy in alleviating pain, restoring function, and preserving joint integrity.

SMO, as a joint-sparing surgical procedure, is now commonly used in early-to-mid-term stages of asymmetric ankle arthritis to correct varus or valgus deformities, adjust alignment, and redistribute the load across the ankle joint, thereby restoring normal biomechanics [20]. Previous studies have indicated that SMO has limited efficacy in patients with ankle arthritis beyond the modified Takakura stage 3a [21]. The clinical efficacy of SMO was also observed in our cohort aligns with existing literature. Our AOFAS improvement (+ 13.7) aligns with Hintermann et al.’s report of + 15.2 points after SMO for malunited pronation-external rotation fractures [8]. Similarly, Christidis et al.’s systematic review documented mean AOFAS gains of 35.4 points across 21 studies, reinforcing SMO’s consistency in functional restoration [9]. Our findings further support recent meta-analyses indicating that SMO remains effective even in Takakura stage 3b arthritis, with low failure rates (6.8%) and moderate revision rates (28.2%) [22]. The correction of the TAS angle (from 80.6° to 90.2° in our study) is consistent with the biomechanical principle of realignment to redistribute joint loading, as emphasized by Barg et al. [15] However, unlike some prior studies [19], we observed that preoperative talar tilt did not correlate with outcomes, a finding corroborated by Collin et al. [23]

Regarding prognostic factors, our analysis revealed no significant correlation between the fracture-to-surgery interval (mean 9.5 years) and functional improvement (AOFAS change, *r* = 0.233, *p* = 0.337), as depicted in Fig. 4. This suggests that SMO can be considered regardless of chronicity, expanding on the work of Lee et al. who also found timing non-prognostic [4]. Furthermore, successful correction of severe talar tilt (preoperative > 7.3°) without compromising outcomes challenges the notion that excessive tilt predicts poor results [19]. These findings reinforce the versatility of SMO across diverse presentations of post-traumatic arthritis, supporting Collin et al.’s conclusion that chronicity does not preclude surgical success [23]. This challenges historical preferences for early intervention.

This study has limitations inherent to its retrospective design, including potential selection bias and unmeasured confounders. The small sample size (*n* = 19) precluded subgroup analyses by fracture type or arthritis severity. Additionally, the absence of a control group limits causal inferences about SMO’s efficacy in halting arthritis progression. Future prospective studies with larger cohorts and longer follow-up are warranted to validate these findings.

## Conclusion

The results of this study indicate that SMO demonstrates favorable early-to-mid-term efficacy in the management of traumatic ankle arthritis caused by fractures, thereby validating our primary hypothesis. Besides, prognostic factors (time since fracture, severe talar tilt) did not adversely affect results, supporting SMO’s utility across diverse presentations. Most patients experienced significant pain relief, functional enhancement, and favorable imaging results, along with high rates of high patient satisfaction. These findings suggest that SMO is a valuable surgical option and warrants further clinical application and investigation.

## Data Availability

All data generated or analysed during this study are included in this article. Further enquiries can be directed to the corresponding author.

## Abbreviations

SMO: Supramalleolar osteotomy
AO: Ankle osteoarthritis
TAS: Tibial anterior surface angle
TT: Talar tilt
AOFAS: American Association of Foot and Ankle Surgery
FFI: Foot Function Index
VAS: Visual Analog Score
CORA: Center of rotation and angulation
